# Medicinal plants of the Bible—revisited

**DOI:** 10.1186/s13002-019-0338-8

**Published:** 2019-11-27

**Authors:** Amots Dafni, Barbara Böck

**Affiliations:** 1 Institute of Evolution and the Department of Evolutionary and Environmental Biology, University of Haifa, Haifa, Israel; 2 Institute of Mediterranean and Near Eastern Languages and Cultures (ILC), Spanish National Research Council (CSIC), Madrid, Spain

**Keywords:** Bible, Medicinal plants

## Abstract

**Background:**

Previous lists number from 55 to 176 plant species as “Biblical Medicinal Plants.” Modern studies attest that many names on these lists are no longer valid. This situation arose due to old mistranslations and/or mistakes in botanical identification. Many previously recognized Biblical plants are in no way related to the flora of the Bible lands. Accordingly, the list needs revision.

**Methods:**

We re-examine the list of possible medicinal plants in the Bible based on new studies in Hebrew Biblical philology and etymology, new studies on the Egyptian and Mesopotamian medicinal use of plants, on ethnobotany and on archaeobotany.

**Results:**

In our survey, we suggest reducing this list to 45 plant species. Our contribution comprises 20 “newly” suggested Biblical Medicinal Plants. Only five species are mentioned directly as medicinal plants in the Bible: Fig (*Ficus carica*), Nard (*Nardostachys jatamansi*), Hyssop (*Origanum syriacum*), balm of Gilead (*Commiphora gileadensis*) and Mandrake (*Mandragora officinarum*). No fewer than 18 medicinal plants are mentioned in old Jewish post-Biblical sources, in addition to those in the Bible. Most of these plants (15) are known also in Egypt and Mesopotamia while three are from Egypt only. Seven of our suggested species are not mentioned in the Bible or in the Jewish post-Biblical literature but were recorded as medicinal plants from Egypt, as well as from Mesopotamia. It is quite logical to assume that they can be included as Biblical Medicinal Plants.

**Conclusions:**

All our suggested Biblical Medicinal Plants are known as such in Ancient Egypt and/or Mesopotamia also. Examination of our list shows that all these plants have been in continuous medicinal use in the Middle East down the generations, as well as being used in the Holy Land today. Precisely in King Solomon’s words, “That which has been is what will be, that which is done is what will be done. And there is nothing new under the sun” (Ecclesiastes 1:9).

## Background

### Identification of Biblical plants

Most of the massive research on the identity of Biblical plant names is based on linguistics and philology [1–8 and references therein]. Włodarczyk [9] reviewed “how many plants are mentioned in the Bible” and concluded that the list contains 206 plant names, 95 of which “are recognized by all contemporary researchers of the floras of the Bible.” This discrepancy is not at all surprising since most authors of books on plants of the Bible [4, 10–17 a except 3, 5–7] were not familiar with Hebrew and/or the Holy Land flora. For example, Duke [1] enumerates at least 176 species as “Biblical Medicinal Plants” (hence BMPs), while the total number of recognized plants in the Bible is about 100 [8]. Needless to say, too many species of his list are not related at all to the flora in the region and were never grown or traded in the ancient Middle East. Jacob [2] listed 55 plants (most on a species level but some on a genus level) as BMPs, based on a comparison to Ancient Egyptian and Mesopotamian literature.

Amar [8] revised the flora of the Bible, based particularly on old Jewish post-Biblical sources and their succession down the generations. He arranged all the traditional plant names in several categories according to identification reliability: (a) plant names identified with certainty (40); (b) plant names identified at a high reliability level (11); (c) plant names whose identification is on a high reliability level but not fail-safe (22); (d) plant names that are unidentifiable or whose identification reliability is very low (13); (e) accumulative names and non-species-specific names like “thorn” or “lily” (20); (f) names suspected of not being related to plants at all (35). Thus, after Amar’s rigorous scrutiny, we have some 75 “valid” plant names, which are regarded with some identification validity. The Mishna and the Talmud mention about 400 plant names [3], 43 of which are mentioned in relation to medicine [18]. In most cases of disagreement among the leading authorities [3–7], we chose to follow Amar’s [8] (see the discussion for few exceptions).

### Identification of Ancient Mesopotamian and Egyptian plants

Plants are undoubtedly the main source for curing and alleviating diseases in Ancient Mesopotamia and Ancient Egypt. Both civilizations belong to the world of the Old Testament, which explains why a short survey about their knowledge of medicinal plants is included. In the strict sense, Mesopotamia refers to the “land between the rivers,” namely the Tigris and Euphrates, but the region includes the area most of now Iraq, eastern Syria and southeastern Turkey. While the first written documents, namely clay tablets, date to the end of the 4th millennium BCE the main information on medicinal plants comes from cuneiform tablets dating to the second and first millennia BCE. Ancient Egypt spans the region of the Nile Valley, reaching areas east and west of it along the Mediterranean coast; to the south, Ancient Egypt stretched deep into the north of modern Sudan. The first hieroglyphic texts on medicine date to the middle of the second millennium BCE. The pharmacopoeia of both cultures included more than 200 plants, most of which cannot be identified. Ethnobotanical studies (e.g. Borchardt [19:190]) often refer to the pioneering work of Campbell Thompson [20] for the identification of Mesopotamian plant terms, or von Deines and Grapow [21] for ancient Egypt; they are unaware of the present, often highly specialized, linguistic and philological discussions in the fields of Assyriology and Egyptology. Philologists such as the Egyptologist Pommerening [22] or the Assyriologist Böck [23] attest to the need to challenge and revise the methodology used so far to identify ancient Egyptian and ancient Mesopotamian plant terms. The *comunis opinio* in both fields of research is rather skeptical about the identification of plant terms with actual plants. In fact, revisions comparable to the comprehensive work of Amar [8] on the Biblical flora are still in process. As for ancient Mesopotamia, identifying language terms in Akkadian, the language in which most of the medical cuneiform texts are written, depends heavily on etymological research. This consists of collecting cognate terms in other Semitic languages such as Aramaic or Hebrew and applying identification of the Aramaic or Hebrew term to the Akkadian name. As a result, learning the identity of Akkadian plants depends basically on studies about the Aramaic and Hebrew terminology of plants (e.g., Löw [3]). These identifications have entered the two basic dictionaries of the Akkadian language [24, 25] but have enjoyed scant discussion and revision.

### The use of medicinal plants in the world of the Old Testament

The ample number of medical recipes prescribing various “drug therapies” clearly shows the prominence of ingredients of vegetable origin in ancient Mesopotamia and ancient Egypt (e.g. [26, 27]).

In the Bible, very few cases related to the use of plants for medicine, for example, the use of balm to treat of sores (Jeremiah, 8, 22; 46, 11; 51, 8) and how King Hezekiah was treated with a fig (II Kings 20:7). Very rarely ethnobotanical information may help concerning the medicinal Biblical plants. An exception is the use of *Origanum syriacum* by the Samaritans in exactly the same manner as in Biblical times [28:71-2]. A few archaeological studies illuminate the use of medicinal plants in the Holy Land in Biblical times and even earlier. Written evidence exists from letters of Tel Al Amarna showing that the King of Gezer (Palestine, 14th BCE) asked for myrrh gum (*Commiphora* sp.) from Egypt for healing [29:29]. Langgut et al. [30] found pollen of three medicinal plants (mint, sage type, and myrtle) in human feces from Megiddo (Late Bronze Age, 12–11th centuries BCE). Langgut et al. consider it (30: 382) “the possible use of different types of herbal teas.” Weinstein-Evron [31] found myrtle pollen in a stone mortar from Megiddo (Iron Age 12–11th centuries BCE). Preparation of powder from *Myrtus* leaves for medicine is also a practice still used today in Israel [32:210-211]. Koh et al. [33] analyzed the organic residues of wine jars found in a courtyard in the Middle Bronze Age (ca 1900–1600 BCE) in a Canaanite palace at Tel Kabri (13 km north of Haifa, Israel). The additives seem to have included honey, Storax resin (*Liquidambar orientalis*), Terebinth resin (*Pistacia lentiscus*/*P. palaestina*), Cedar oil (*Cedrus libani*), Cyperus (*Cyperus rotundus*) and Juniper (*Juniperus communis*/*J*. *phoenicea*), and perhaps even mint, myrtle, or cinnamon. They concluded that “the plants’ materials were used to preserve wine (as resins), as well as a medicine already known from Ancient Egypt” [34]. They also mentioned that “these additives suggest a sophisticated understanding of the botanical landscape and the pharmacopeic skills necessary to produce a complex beverage that balanced preservation, palatability, and psychoactivity.” Namdar et al. [35] found cinnamon residues in old wine flasks from Tel Dor (30 km south of Haifa, Israel). The flasks, originating in Phoenicia, were from the early Iron Age, namely 11th to mid-9th centuries BCE. Kislev et al. [36] studied the remains of flax (*Linum usitatissimum*) from the early Iron Age (12th century BCE, late 20th dynasty in Egypt) site of Tel Beth-Shean (70 km SE of Haifa, Israel). Like written sources of its uses, they suggest that part of the flax seeds was intended as a food component or for extracting medicinal oil. Weiss and Kislev [37] found one stone of bay laurel (*Laurus nobilis*) in Ashkelon (150 km south of Haifa, Israel l7th century BCE). The plant does not grow in that area, so the plant was probably taken for medicinal purposes.

### Magic and medicine in the world of the Old Testament

In the ancient world, there is no clear-cut distinction between ritual/magic and medicinal uses of the same plant (especially incense) [38:12, 37: 39 passim, 40: passim, 41: passim]. Medical practices in Egypt [41: passim] and in Mesopotamia [42: 415-425] involved, in addition to the use of medicinal plants, rituals and incantations.

The Ancient Egyptian and Mesopotamian healing practitioners did not contrast or distinguish magical from pharmaceutical cures—both forms of healing were considered equally effective (for Egypt see 43; for Mesopotamia see 44). The two ancient cultures shared another feature: they did not invent terms that would denote “medicine” or “magic.” Disease and illness could be caused by an array of incidents from natural causes to the supernatural influence of deities and demons or sinful behavior (for Egypt see 41: 96-112, for Mesopotamia see 45: 30-31). The healers seem not to have chosen their cures according to the cause of the disease. It is useful to differentiate healing cures accompanied by incantations from purely exorcist practices. In Mesopotamia, demons were deemed responsible for diseases [44: 179-180, 45: 27-39,] while in Egypt, it was evil spirits [41: 96-112]. Thus, healers also practiced magic and exorcism as part of the healing. In the Bible, magic and exorcism were forbidden [46:517-519]. In Egypt and Mesopotamia, sin was believed to be sanctioned with disease [47: 97-99]. Krymow [48:16] has noted that “The Israelites knew the medical practices of the Egyptians and took this knowledge with them, but the Israelites’ priests taught the people to look to God for help.” A similar view is expressed by Harrison [38:14]: “The religious tradition of the early Biblical period took exception to the idea of trying to cure the diseased body, since it was believed that God alone was the great healer.” Our paper, set out to re-examine the list of possible medicinal plants in the Bible based on new studies in Hebrew Biblical philology and etymology, and on new studies on Egyptian and Mesopotamian medicinal use of plants, ethnobotany, and archaeobotany. Special attention is given to the history and the knowledge of medicinal uses for indigenous flora of the Holy Land and the ancient trade in plants and their products.

This paper does not aim at a detailed catalogue of specific uses for each plant in each civilization. Our study is limited to re-examining questions arising from the list of actual and potential medicinal plants of the Bible.

## Methods

Working assumptions and problems in the identification of Biblical plant names:

1. Some problems regarding the identity of Biblical plant names originated from misunderstandings of the original Hebrew version in which many plant names are not clear. A new study of the flora in the Old Testament [8] provides new scope concerning plants mentioned in the Bible, while assessing the reliability of all previously suggested botanical identifications of plant names. Plant names in the New Testament have been revised in recent dictionaries, e.g., Greek-English Biblical dictionaries [49, 50] and translations [e.g., 51]. Similar problems arose over modern references concerned with plants in the Talmud [18, 52, 53].
2. The same plant may have several names even in the same country [32 passim, 44: 132, 54:43, 55:7, 56:51]. The same name of a plant may refer to more than one botanical species and/or genera [55-58: passim]. Plants that are heavily used in medicine and witchcraft tend to have many local names (e.g., Mandrake [57]). Plant names may change down the generations; some old names may be discarded or forgotten even in the same language [58:520].
3. The old translators of the Bible, e.g., King James Version (1611 and others, see 4:7-11), were not familiar with the original Hebrew, nor with the flora of the Holy Land. So, sometimes, they mentioned names from their local floras; this might also have been done deliberately to make the plants more familiar to their own readers.
4. In general, the Bible does not refer directly to plants, most of which are mentioned in passing. The chances that a specific plant would be connected directly with a medicinal use are even lower. Linguistic remains, ethnobotanical as well as archaeobotanical, may help, but they are not evidence of possible specific medicinal uses.
5. When studying plants not mentioned in the Bible but in the Talmud, in a medicinal context, we have to remember that Talmudic medicine may have Hellenistic and Egyptian influences [59: xiii, 53:29-31]. If these plants are also recorded as medicinal plants from Mesopotamia, this may reduce that kind of bias.
6. New works, especially on the identification of Assyrian plant names [24, 56, 44:129-163, 60] considerably extend the spectrum of validating plant names and changing previous conceptions. All previous works on Biblical plants [e.g., 2, 4, 6-8, 15, 16] were based solely on Campbell-Thompson [20], who had been highly criticized [61:492, 62:3, 63:326]. Jacob [2] was criticized by Geller [63:326] because he “assumes that the existence of a plant is sufficient to identify it within the Egyptian and Akkadian pharmacopeia, entirely ignoring the considerable philological problems in such methodology.”
7. It is logical to assume that plants (or their products such as spices and incense), which had medicinal uses in Egypt and Mesopotamia, were also known in the Holy Land in Biblical times, even if these plants are not mentioned directly in the Bible [2:29, 64:69-70]. Cultivated plants (or their products), which are mentioned in the Talmud as medicinal plants and are also documented in Egypt, Mesopotamia and/or from archaeological evidence, are considered to have been present in the Holy Land in Biblical times. This approach is based on the evidence of intensive ancient use of and trade in medicinal plants all over the Fertile Crescent [19:188, 64:69]. Remember too that some medicinal plants were introduced into Egypt by way of Palestine [64:71]. Manniche [65: 61] pondered how to decide whether or not a certain Egyptian species was really a “medicinal plant.” She concluded: “The actual remains of a plant…must be supported by some indication of the use of plant—ideally—in the Egyptian texts; in texts from contemporary neighboring civilizations ….” In Palestine, plant remains are quite rare (compared with Egypt); hence, a comparison with other contemporary cultures from the Bible period is of prime importance when considering the medical use of a given plant species”.

Procedures: 1. Checking the validity of the identification of the medicinal plant names in the Bible according to Amar ([8] see above). We discard all previous lists of plant names that were supposedly mentioned in the Bible based on old mistranslations [see 66]. Many of these are not indigenous to the Holy Land at all or were never introduced.

2. Reconstruction of the inventory of potential BMPs was attempted, based on comparative data from Ancient Egypt and Mesopotamia. The medicinal plants of Egypt and Mesopotamia were surveyed, keeping with the recent literature, in an attempt to recognize species, or products thereof, related to Biblical times. We limited ourselves to any literary evidence that a certain species had any medicinal use; we did not set out to compare the different regions/cultures on the specific uses among them.

3. We also used complementary data from post-Biblical sources: Mishna (3rd century CE) and the Babylonian Talmud (3rd–5th centuries CE). We considered only plants referred to explicitly for medical uses and already known medicinally from Egypt and/or Mesopotamia, and/or from archaeological evidence. Concerning the identification of plants in the Talmud, in cases of disagreement, we followed the most modern commentary of Steinsaltz [67] (whose botanical advisor is the authoritative archaeobotanist and Talmudist M.E. Kislev). As a result, several of the previous identifications [3, 18] are not recognized today.

4. Technically, we divided the surveyed plants into four classes, according to level of certainty as to their possible use as medicinal plants in Biblical times, based on identification reliability according to [8], as well as on subsidiary evidence: plants used or mentioned explicitly as medicinal in the Bible (Table 1); plants mentioned in the Bible and known as medicinal in Ancient Egypt and in Mesopotamia (Table 2); plants not cited in the Bible but mentioned as medicinal in post-Biblical sources and/or Egypt and/or Mesopotamia (Table 3); and various patterns (Table 4).

**Table 1:** Plants used or mentioned explicitly as medicinal in the Bible

Species /English name	Hebrew name /transcription /level of identification reliability	Source of evidence	Botanical status/ Origin/	Additional uses:
*Commiphora* *giledadensis* (L.) C. Chr/ (or *C. kataf* (Forssk.) Engl. */* (also *C.* *opobalsamum* (L.) Engl.) Balm of Gilead, Balm of Mecca	צרי Tsori C נטף Nataf B	**E:** [68:85 (*C.* *myrrah*); [69 passim (myrrh), [64:77, 70:63]. **B:** Jeremiah 8:22, 46:11, 51:8 **M:** [71:193, 72:89,90, 73:5].	D / Im/ EAf [73a]. Cultivated in the Holy Land at Biblical times [74].	In (Exodus 30:31) / O / P
*Ficus carica* L.* / Fig	תאנה Te’enah A	E: [64:78, 65: 102-103, 75:28]. B: II Kings 20:7, Isaiah 38:2. BT: Menachot 64a. M: [44:430,437].	D / In	E
*Mandragora* *officinarum* Mill * / Mandrake	דודאים Duda'eem A	**B**: Genesis 30: 14- 17. **E**: [65:118] (possible**).** **M**: [71:191, identification not ascertained	In	M / A
Nardostachys jatamansi DC. / Spikenard	נרד Nerd A	**NT:** Mark 14:3; John 12:3.	EA [76] / Im	O /P
*Origanum* *syriacum* L*.* *(=Majorana* *syriaca* (L.) *Feinbrun*) / Syrian Marjoram / Bible Hyssop	אזוב Ezov A	B: Leviticus 14:4, Psalms 51:9. BT: Shabbat 109b, Abodah Zara 29a.	In	R / E

Botanical status: *In*: Indigenous; *Im*: Imported; *D*: Domesticated/cultivated. If there is no specific reference, we followed [143].

Origin: *A*: Arabia; *CEA*: Central East Asia; *E*: Europe; *EAR*: East Arabia; *EA*: East Asia; *EAf*: East Africa; *M*: Mediterranean; *EM*: East Mediterranean; *NE*: Near East; *SEA*: Southeast Asia; *SEE*: Southeast Europe; *SWA*: Southwest Asia. If there is no specific reference, we followed [143].

Additional uses: *Ap*: Apotropaic; *E*: Edible; *F*: Fibre; *I*: Incense; *M*: Magic; *P*: Perfume/cosmetics; *O*: Ointment; *Oi*: Oil; *R*: Ritual; *S*: Spice, condiment; *Re*: Resin; *T*: Timber; *W*: Wine

*Included in Jacob’s [2] list (see text). The categories of the reliability levels of the Biblical plants’ identification are according to Amar [8]. See above

**Table 2:** Plants mentioned in the Bible and known as medicinal in Ancient Egypt and Mesopotamia

Species / English name	Hebrew name / Transcription / Level of identification reliability	Source of evidence	Botanical status / Origin	Additional uses
*Acorus* *calamus* L.* / Sweet reed	קנה Kaneh / קנה הטוב Kaneh hatov / קנה בשם Kneh Bosem C	E: [65:68]. M: [20:10].	Im / EA [77].	P
Allium cepa L.* / Onion	בצל Batsal B	**E:** [64:78, 65:69, 70:85]. **MI:** Nedarim 9:8. **BT**: Nedarim 66a. **M**: [78:386, 79:20].	D (CEA)	E
*Allium porrum* L. * / *A. kurrat* L*.*	חציר Khatsir A	**E:** [65:70, 69:96]. **BT:**Yoma 83b, Berachot 44b**,** Gittin 69ab, Shabbat 110b **M**: [79:20, 80:234,].	D / M- EWA	E
*Allium sativum* L. / Garlic	שום Shum A	**E**: [64:78, 65:70-71, 68:85, 71:38,42,70,80,88, 75:28]. **BT:** Gittin 69a, Abodah Zara 28b. **M:** [78: passim, 81:123,127].	D (SWA-CA).	E
*Anethum* graveolens* Dill	**NT**: (Matthew 23:23. = Anise *(Pimpinella* *anisum* L. in King James Version. Dill (*Anethum* *graveolens* L.) in modern translation [51:108].	**E**: [64:71; 68:85, 70: 99,372,373,375].	D / E	E BT: Abodah Zara 7:72, Niddah 51a
*Artemisia* sp.	לענה La'anah C	E: [65:80] (A. absinthium). M: [78 passim] (as “wormwood”) BT: Abodah Zara 29a.	I	
Boswellia* sp. / Frankincense	לבונ Levonah A	**E:** [68:85, 70:69,373,375 (*B. arterii*)]. **BT:** Sanhedrin 43a. **M**: [20:344, 82:251] (identification not ascertained).	Im (EA)	I
*Capparis* *spinosa* L.	צלף Tsalaf C	**E**: [65:83]. **BT:** Shabbat 110a **M**: [71:191].	In	E
*Cedrus libani* A. Rich.*/ Cedar */*	ארז Erez A	**E:** [64:6, 69:88 75:26]. **AR**: [33]. **M:** [81:124] (oil); [71:191,193, 72:89, 73:52, 78:52,78, 80:233,235, 81:121, 83:124] (resin).	Im (Lebanon; **B**: II Samuel 5:11).	Re
*Cinnamomum* *zeylanicum* Nees* *and C. cassia* Blume \	קינמון Kinnamon C	**E**: [65: 88-91]. **AR**: [35, 33(?)]. **M**: [20:189-190].	Im (EA).	S
*Citrullus* *colocynthis* (L.) Schrad. / Colocynth, Bitter gourd	פקועה Paqqu'a A	**E**: [65:91,68:85]. **M:** [81:122,124, 125].	In	Oil (**BT**: Sabbath 71:26)
*Citrus medica* L.* /Citron	פרי עץ הדר Pri Ets Hadar A	**BT:** Shabbat 109b**.** **AR:** 5th - 4th centuries BCE, [84, 85]. **M:** [20:312] (Identification not ascertained).	Im (EA)	R
*Commiphora* sp*. /* Myrrah / Myrrh	מור Mor B	**E:** King of Gezer (Palestine, 14th BCE) asked for myrrh gum from Egypt for Healing [29:29]. **M**: [86:156-157].	Im / EAr [73a]	I / R / O / P
*Coriandrum* *sativum L. /* *Coriander*	גד Gad A	**E:** [64:71, 65:94-95, 69:42,82]. **M:** [20:66, 71:192, 73:52, 78:283].	In / D (At the Talmud period: Shabbat 133a, 134a).	S
Crocus sativus* L./ Saffron	כרכם Karkom A	**E:** [75:30, 69:42]. **BT:** Shabbat 110a, b, Gittin 69a. **M:** [73:52, 71:191].	Im (SWA)	S / I
*Cuminum* *cyminum* L. / Cumin	כמון Kammon A	**E:** [64:71; 65:96-98]. **BT:** Shabbat 110a,b, 133a, Gittin 69a,b, Abodah Zara 29a. **M:** [80:234, 78: Passim, 81:123].	D /SWA	S
*Cupressus* *Sempervirens* L. * / Cypress	ברוש Brosh C	*Juniperus:* **E**: [64:29,72, 69:42,71,70,72,81 ,88,80, 75:28] (berry). **AR:** [31] *(J. phoenicea /* *communis*) **M:** [80:233,234,235, 87:26], (wood) [73:52-**,** 53, 83:120], (*burasu*); 68:19, 87:26,27]. *Cupressus:* **M:** [80:233] (oil) [68: 191,193].	In / I	
*Juniperus* sp.*/Juniper	ערער Ar'ar C			
Hordeum* sp. / *Barley*	שעורה Se'orah B	**E:** [70:140ff, 372, 375]. **BT:** Gittin 69b, Shabbat 110b, Pesachim 42b, Yoma 83b.	D	E
*Linum* *usitatissimum* L. * Fl*ax*	פשתה Pishta A	**E**: [65:116, 75:28; 70: 57– 62]. **AR**: [37] (?). **M:** [20:113].	D/MED-SEA.	F
Mentha* spp. Mint	NT: Matthew 23:23.	**E**: [65:120; 75:28,29, 68:85; 69:66,86]. **AR**: [30 (?); 33(?)]. **BT:** Sabbath 140a, Gittin 69b, Abodah Zara 29a (as "Ninia"). **M**: [81:120,126, 78 passim, 89:50].	In / D	S
*Myrtus* *communis* L.* / Myrtle	הדס Hadas A	**AR:** 12-11th century BCE, [30]; 12th-6th centuries BCE [31]. **M**: [20:300, 73:52, 80:233, 87:26].	In	R /Ap
*Nigella sativa* L.* / Black cumin	קצח Ketsakh K	**M**: [90: 268, 91:223].	D/ SWA	S
*Olea europaea* *L. / Olive*	זית Zay'it A	**E**: [65:128-129]. **BT:** Gittin 86a; Shabbat 134a; Abodah Zara 28a, Berachot 36a. **M:** [79:18].	In.	E / Oil
*Phoenix* *dactylifera* L.* / Date Palm	תמר Tamar A	**E**: [68:83,84, 69:41,71,81]. **BT:** Ketubbot 10b, Gittin 69a, Shabbat 110a.	In (SWA) / D (**BT**: Ketubot 10b, Gittin 69b, 70a, Yoma 84a, Sabbath 109b.	E
	דקל Dekel A			
*Punica* *granatum* L.* / Pomegranate	רימון Rimmon A	**E**: [64:77, 65:183-141, 75:29]. **M:** [78 passim, 80:235].	D (SWA-SEE)	E / R
*Ricinus* *communis* L. */ *Castor Bean* *(see* *discussion)*	קיקיון Kikkayyon C	**E**: [64:79, 65:142-143, 69:41,70,71 70:119,122,333, 373, 375]. **BT**: Gittin 69b (as "*Akika*"). **M**: [20:130].	D /EAf [92] BT: Cultivated at the Talmud period. Sabbath 21:71	Oi
*Sinapis alba* L*.* Mustard (Could be also *Brassica* *nigra* L*.*) */* Mustard	Khardalחרדל	**E***:* [65:148, 68:85(?)]. **BT:** Berachot 40a **M:** [70:90, 78:367,368, 93:142].	In / D	E / S
*Vitis vinifera* L. * / Grape	גפן Geffen A	**E:** *Fruit*: [70: 85,372,374]; *Wine*: [70:91]. **BT**: *Wine*: Shabbat 78a, 109ab,110b, Gittin 67b; Ktubot 65b. *Vinegar*: Eruvin 29b; **BT:** Shabbat 109a **M**: [79: 28].	D / EM-WA	E / W

Botanical status: *In*: Indigenous; *Im*: Imported; *D*: Domesticated/cultivated. If there is no specific reference, we followed [143].

Origin: *A*: Arabia; *CEA*: Central East Asia; *E*: Europe; *EAR*: East Arabia; *EA*: East Asia; *EAf*: East Africa; *M*: Mediterranean; *EM*: East Mediterranean; *NE*: Near East; *SEA*: Southeast Asia; *SEE*: Southeast Europe; *SWA*: Southwest Asia. If there is no specific reference, we followed [143].

Additional uses: *Ap*: Apotropaic; *E*: Edible; *F*: Fibre; *I*: Incense; *M*: Magic; *P*: Perfume/cosmetics; *O*: Ointment; *Oi*: Oil; *R*: Ritual; *S*: Spice, condiment; *Re*: Resin; *T*: Timber; *W*: Wine

*Included in Jacob’s [2] list (see text). The categories of the reliability levels of the Biblical plants’ identification are according to Amar [8]. See above

**Table 3:** Plants not cited in the Bible but mentioned as medicinal in post-Biblical sources and/or in Egypt and/or Mesopotamia

Species / English name	Source of evidence	Botanical status / origin/	Additional uses
*Aloe vera* L. * Aloe	**E**: [65:72, 75:25-26 , 68:82,83,84,85]. **BT**: Gittin 69b. **M**: [20:129, 94:533].	D /Ar [99].	C
*Carthamus tinctorius* L. / Safflowers	**E**: [64:79, 65:83-84, 69:79]. **BT:** Gittin 70a (? As "*Morika*") **M:** [96: 544-545]**.**	D / NE [97].	E / Oi
*Ferula asa-foetida* L. / Asafoetida	**E**: [65:101-102]. **MI:** Shabbat 20:3. **BT:** Shabbat 140a; Gittin 69a (under *Helbena*) **M:** [20:355, 73:52, 83:11].	CA [98]	In
*Lawsonia inermis* L*.* Henna	כפר Koffer A **E:** [65:114, 68:85].	Im / EA	D: **BT**: Shevi‘it7:6. P **B**: Song of Songs 4:13).
*Lepidium sativum* L. / Cress	**E:** [64:71, 65:115]. **BT:** Shabbat 110a, Yoma 49a, Gittin 69a. **M:** [72:89,90,91 73:52,53, ].	D / SA (perhaps Iran, [99]). **BT**: Gittin 69a, Shabbat 109a.	E
*Piper spp./* Black pepper	**E:** [64:71, 65:136]. (*Piper nigrum*). **BT:** Shabbat, 65a, 90a, 140a]. **M:** [83:120].	Im / EA [99].	S

Botanical status: *In*: Indigenous; *Im*: Imported; *D*: Domesticated/cultivated. If there is no specific reference, we followed [143].

Origin: *A*: Arabia; *CEA*: Central East Asia; *E*: Europe; *EAR*: East Arabia; *EA*: East Asia; *EAf*: East Africa; *M*: Mediterranean; *EM*: East Mediterranean; *NE*: Near East; *SEA*: Southeast Asia; *SEE*: Southeast Europe; *SWA*: Southwest Asia. If there is no specific reference, we followed [143].

Additional uses: *Ap*: Apotropaic; *E*: Edible; *F*: Fibre; *I*: Incense; *M*: Magic; *P*: Perfume/cosmetics; *O*: Ointment; *Oi*: Oil; *R*: Ritual; *S*: Spice, condiment; *Re*: Resin; *T*: Timber; *W*: Wine

*Included in Jacob’s [2] list (see text). The categories of the reliability levels of the Biblical plants’ identification are according to Amar [8]. See above

**Table 4:** Various patterns

Species / English Name	Source of evidence	Botanical status / Origin	Additional uses:
*Ceratonia siliqua* L*.* * Carob	**M:** [20:175,180,184,186,284, 100:41].	In / D: **BT**: Baba Batra 68b.	E
Eruca sativa* Mill. */* Rocket See discussion	**BT**: Yoma 18b, Shabbat 109a, Gittin 69b (under “*Gargir*”). **M:** [20: 210].	D, **BT**: Eruvin 28b, Ma'aserot 48:73.	E
*Foeniculum vulgare* Mill. C	**E:** [65:105-106, 75:28, 71:41] **M:** [73:52, 101: 234-242].	In	E / S
*Laurus* *nobilis* L.* / Bay Leaf*,* Laurel See discussion	**AR**: [37]. **BT**: Gittin 69b	In	S
*Pistacia lentiscus* L.* / Lentisk	**E**: [70:80]. **AR**: [33], (*P. lentiscus /P. palaestina*). **MI**: Tosefta Shabbat 12:8.	In	R
*Sesamum indicum* L*.* Sesame	**E:** [64:79, 65:147, 68:85]. **BT:** Gittin 69b. **M:** [80:233].	D / SEA **BT**: Very common crop in the Talmud period, Sabbath 89:42, Nedarim 86:49, **MI**: Machshirin 81:46.	E, **BT**: Sabbath 82:42, Nedarim 86:49. / Oi
*Trigonella foenum –graecum* L. / Fenugreek	**E:** [65:85-87, 68:85]. **M:** [73:52-53, 78:430**]**.	In /EM-SWA / D	E

Botanical status: *In*: Indigenous; *Im*: Imported; *D*: Domesticated/cultivated. If there is no specific reference, we followed [143].

Origin: *A*: Arabia; *CEA*: Central East Asia; *E*: Europe; *EAR*: East Arabia; *EA*: East Asia; *EAf*: East Africa; *M*: Mediterranean; *EM*: East Mediterranean; *NE*: Near East; *SEA*: Southeast Asia; *SEE*: Southeast Europe; *SWA*: Southwest Asia. If there is no specific reference, we followed [143].

Additional uses: *Ap*: Apotropaic; *E*: Edible; *F*: Fibre; *I*: Incense; *M*: Magic; *P*: Perfume/cosmetics; *O*: Ointment; *Oi*: Oil; *R*: Ritual; *S*: Spice, condiment; *Re*: Resin; *T*: Timber; *W*: Wine

*Included in Jacob’s [2] list (see text). The categories of the reliability levels of the Biblical plants’ identification are according to Amar [8]. See above

## Results and discussion

## Discussion

Only five species (Table 1) are mentioned explicitly as medicinal plants in the Bible: Fig (*Ficus carica*), Nard (*Nardostachys jatamansi*), Hyssop (*Origanum syriacum*), “Balm of Gilead” (*Commiphora* sp.) and Mandrake (*Mandragora officinarum*) (Table 1). Twenty-seven species come under the category “Plants which are mentioned in the Bible and are known as medicinal in Ancient Egypt and Mesopotamia” (Table 2). Thirteen species are included as “Plants which are not cited in the Bible but mentioned as medicinal in the Talmud and/or Egypt and/or in Mesopotamia” (Table 3). Six plants are classified under various patterns (Table 4).

At least 18 medicinal plants (Tables 2–4), in addition to those in the Bible, are mentioned in the Talmud and or Mishna, most of which (15) are known also in Egypt and Mesopotamia, while three are only from Egypt. Since most of the post-Biblical citations are from the Babylonian Talmud, one may consider it as having influenced the local medicine of Babylonia (where this Talmud was written) rather than reflecting Biblical reality. The data indicating that all these species were also known from Egypt, strengthen the idea that the post-Biblical literature was not biased to Mesopotamian plants.

Seven of the suggested species (Table 4) are not mentioned in the Bible or in the Talmud but were recorded as medicinal plants from Egypt, as well as from Mesopotamia; it is logical to assume that they can be included as BMPs.

About 60% of our suggested BMPs are foreign species; 40% are indigenous, 60% are imported, 30% are domesticated (each plant can belong to more than one group). The main sources of foreign imported species are East Asia (11%), Southwest Asia (11 %), West and South Asia (8%) and Arabia (4%).

The high proportion of imported plants (as medicinal materials) shows indirect evidence of prolific import in Biblical times [101–104]. Our list does not provide any evidence that any species were cultivated/imported solely as medicinal plants; all had some additional use. Most (87%) of the species had at least one additional use: for example, 16 are edible, 8 are used in rituals, 6 serve for perfume and cosmetics, and 5 are used as incense.

Duke [1] enumerated 176 plant species as “Biblical Medicinal Plants,” while Jacob [2] suggested only 55. In our survey, we suggest reducing that number to 45 (Tables 1-4). The overlap between Jacob’s list and ours was 29 species in total. Our contribution is 20 “new” suggested BMPs. It is noteworthy that some Biblical names are related to the genus level (e.g., *Artemisia*), or also to two genera as in the case of *Cupressus/Juniperus*.

The discrepancy between Jacob’s list and ours is due to the following: (1) At least 22 species in Jacob’s list are not recognized today as valid “Biblical plants” at all, or they are not related to any specific plant species or genus [8]. (2) Several identifications from Campbell–Thompson [20], the only Mesopotamian source used by Jacob, are no longer recognized by modern Assyriologists. (3) Several Mesopotamian plants were only recently identified in a medical context. (4) New recent palynological as well archaeological data allow us to corroborate the possibility of Biblical medicinal uses of some plants.

We excluded about 24 plant species from Jacob’s list [2] for the following reasons: they are no longer recognized by Amar [8] as plants, or the reliability of identification level is below “low probability of identification” or is an “aggregate name” and not a species-specific plant name (hence, indicated as NR); the identification by Campbell–Thompson is no longer recognized today or cannot be confirmed by any further evidence (NC-P); the plant is unknown to the flora of the Holy Land (NF); no evidence exists of international trading in the plant (NT); identification is obscure and/or non-specific (OB); they are recorded only from Egypt, with no further evidence related to the Holy Land (OE); they are found only in Mesopotamia (OM), with no data from Egypt and/or Mesopotamia on any medicinal use (EM).

These plants are (1) *Zizyphus vulgaris* Lam. [20: 319: ff; NF], *Z. spina christi* (L.) Wild. 65:158 (OE). (2) *Papaver rhoeas* L. (based on [70:327]). While Manniche [65:130-132] and Aboelsoud [68:85] mentioned that *P. somniferum* L. was used medicinally in Egypt, Bisset et al. [105] could not corroborate this information. It is also debatable whether *P. somniferum* was known at all in the Holy Land in Biblical times [106-108]. (3) *Nymphaea coerilea* (sic!), *Lotus* L (sic!), based on Germer [70:26, 373, 375] (NF). *Lotus* sp. (NF, NT) appears in Manniche [65: 126-7] under *Nymphaea lotus* (L.) Willd. (See 70:64, 26: 373, 375), (NF, R, NT). (4) *Anemone coronaria* L. (NR); (5) *Anthemis nobilis* L., not in Egypt [65], mentioned for Mesopotamia [20:117] (NF, NR, NT); (6) *Colchicum autumanale* L., [20: 167] (NR, NF, NT). (7) *Ranunculus* sp., [20: 146, not in 65]. Twenty-four species of *Ranunculus* are known from Israel [109:192-198], not one of which is known locally as a medicinal plant [110,111]; (8) *Urtica dioica* L., [20: 209], (NR, NF, NT); (9) *Quercus infectoria* Oliv. , [20:2470, (NF, NT); (10) *Nerium oleander* L. (not in Manniche [65]), mentioned for Mesopotamia [20:322]) (NR, NT); (11) *Lolium temulentum* L./ (Matthew 13:25), Schonfield [51:85] translated it as “weed” and added in note 21 “Wheat-like weeds (Heb. Zunin, Gr. *zizania*), probably darnel.” In the King James Version, it appears as “tares,” which is identified as *L. temulentum* [4:133-134], mentioned for Mesopotamia [20:148] (OM; (12) *Triticum* sp. (Emmer wheat) [65:152-3], Gittin 69b (EM); (13) *Prunus amygdalus* Batsch (= *Prunus dulcis* (Mill.) A.A. Webb) [70:224; 65:138-139], (OE); (14) *Balanites aegyptiaca* (L) Delile, according to Manniche [65:81], “Its use remains somewhat obscure” (NR); (15) *Phragmates communis* (sic!) (the valid synonym of *Phagmites communis* Trin (= *P. australis* (Cav.) Trin. Ex Steud.) based on Germer [70:188,190] not in [65], (OE); (16) *Chrysanthemum cinerariifolium* (Trevir.) Sch. Bip., based on Germer [70:263] not in [65], (NR, NF); (17) *Arundo donax* L. [70:188,191) not in Manniche [65] (OE, NT); (18) *Nymphaea lotus* L. [70: 26, 373, 375] (NF); (19) *Cucumis melo* L. [70:124,373,375, 65:76] var. *chate* (Hasselq.) Sageret (OE, NT). *Cyperus papyrus* L. [70:138,187,201,373,397, 65: 100] (OE, NT); (20) *Cyperus esculentus* L. [65:98, 70:134,201,207,222,372,375] (OE, NF); (21) *Thymus vulgaris* L. known from Egypt [70:17] (NR); *Thymus* sp. in [65:150] (NR, OE, NF); (22) *Citrullus vulgaris* Schrad. [70:266)] (OE); (23) *Salix safsaf* Forssk. ex Trautv. [70:106,237,373; 65:145-76; 75:30; 69:42], willow buds (OE, NF); (24) *Ficus sycomorus* L. [75:30; 69:41,79, 82] (OE).

The following additional medicinal plant species are known only from Ancient Egypt; at the moment, no sufficient supporting data exist to consider them BMP’s: *Glycyrrhiza glabra* L. [65:106]; *Portulaca oleracea* L. [65:13 7-138]; *Raphanus sativus* L. [65:141-142]; *Rubia tinctoria* Salisb. (=*Rubia tinctorum* L.) [65:144]; *Acacia nilotica* (L.) Delile [68:84; 65:65-67; 69:79, 88, 91, 92, 95, 97]; *Acacia* sp. [69: 39, 41, 42 gum, 88]). *Ocimum basilicum* L*.* [65:128; 70:84]; *Cannabis sativa* L. [65:82].

*Debatable species:* “*Ohalim / Ohalot*”. In the Bible “*Ohalot*” and “*Ohalim*” are mentioned four times—three of them in relation to perfumes (Proverbs 7:17; Psalms 45: 9 and Song of Songs 4: 14). Amar [8: 156-7] concludes that the old Jewish interpreters agree only on the identification of “*Ohalim*” cited in Songs of Songs as *Aquilaria agalocha* Roxb. (= *A. malaccensis* Lam.). According to Amar [8:156], this identification is on a level of “high probability but not certain.” Felix [6:255] considers all the citations related to “*Aquilaria agallocha.*” But Zohary [7:204] considers only the Psalms citations, as well as John (19:309-40), as related to *Aquilaria agallocha* /Aloe vera. In the Talmud (Gittin 69b), “*Illava*” is mentioned as a medicinal plant. It is held to be Aloe vera by most of the old Jewish commentaries [4, I:150].

“*Oren*”—*Pinus* or *Laurus nobilis*? Amar [8:158] takes “*Oren*” (Isa. 44:14-15) to be *Pinus halepensis* Mill., under “A high level of the identification reliability but not sure.” Cedar in Akkadian is “*Erenu*” [112: 181-182]. In Campbell–Thompson’s view [20:282], “*erini*” or “*erinu*” is used as a general term for the coniferous tree, a view not accepted today. In the Talmud (Gittin 69b), there is a remedy called *Atarafa d*’*ara*’*a*” (אטרפא) against stomach worms; “*tarfa*” means a leaf and “*de*’*ara*’*a*” is translated by A. Steinsaltz [67] (in his commentary to Gittin 69b) as *Laurus nobilis*, based on the name of this species in other Semitic languages as “*ar.*”

Campbell–Thompson [20:298] mentions “ēru” (which he identifies as *L. nobilis*) against “anus troubles”; this identification cannot be confirmed or denied because of the lack of sufficient cuneiform evidence. Therefore, Feliks’s [6:92] and Zohary’s [7:120] acceptance of “*Oren*” as *Laurus* has no solid evidence. It is worth mentioning that, as a rule that Campbell–Thompson’s [20] identification is based mainly on Aramaic and Hebrew terms. So, following Campbell–Thompson, to clarify the Biblical plant terms, this might end in a vicious circle!

*Ceratonia siliqua*. Despite the debate over whether *Ceratonia* is mentioned in the Bible [3, I: 393-407, 4:72-73; 113:passim, 114: passim], the plant clearly was widely distributed in the Holy Land as an indigenous species [115]. The few archaeological findings of *Ceratonia* as: phytolites [116:1259]; wood [113:85; 117:112]; seed and fruits [118:101; 37:4] as well as pollen [119:12,18] indicate its presence in the Holy Land in the Bible period and earlier. All of the authorities agree that it was present here naturally, even if it is not mentioned directly in the Old Testament [113, 114, 116, 117 and references therein]. There is a debate [120: specimen No. 41; 4: 72-73; 7:63 and reference therein] if the “Locust” cited in Matthew 3:4 and the “pods” of Luke 15:16 are really *Ceratonia***.** The many Jewish post-Biblical references indicate its high importance as a food plant in the Holy Land [121:203-204; 3, II: 393-407]. Indeed, the presence of the carob in the Holy Land during the Bible period is quite certain; it is a common medicinal plant in the region [rev. 122].

“*Brosh*”–*Cupressus/Juniperus*: Amar [8:159-161] discusses in detail the different Jewish historical suggestions for the identification of “*brosh*,” and summarizes: “it seems that we are speaking on an aggregate name for both genera *Cupressu*s and especially *Juniperus*.” Notably, Löw (*Cupressus*—[3, II:26-33] *Juniperus*—[3, II: 33-38]) and Felix [6: 79-80] have the same view. According to Zohary [7:106], “It probably refers to *Abies cilicica* (Antoine & Kotchy) Carriére. Today, there is general agreement that the Akkadian word “*burashu*” denotes Juniper [112: 180-181). The Akkadian term for cypress is “s*hurmenu*” [112: 184]. Some of the previous ideas on this issue [123–125] are not accepted today.

“*Kikkayyon*”–*Ricinus*/*Lagenaria*: Amar [8: 178-179] following [126: 352-354] suggests that the identification of the Hebrew “*Kikkayyon*” as *Ricinus communis* is of a “high but not sure level of identification's reliability” and that it could also be *Lagenaria vulgaris* Ser. ( = *L. siceraria* (Molina) Standl.). Although *Lagenaria* is known as a medicinal plant [127], it is not as common a medicinal plant as *Ricinus*. Felix [6:136] mentions the possibility of *Lagenaria* being included, based on some old Jewish sources. He states that “the Talmudic tradition identifies ‘Kikkayyon’ as *Ricinus* based on philology and the Geonim (the presidents of the great Babylonian Talmudic schools) evidence that this plant is common in Babylonia.” Zohary [7:193] did not mention the possibility of *Lagenaria* at all. We prefer to relate “*Kikkayyon*” to *Ricinus* because of its wide use as an important medicinal plant in the ancient world since ancient times [rev.128], including the Talmudic period (Shabbat 21a).

“*La*’*ana*”–*Artemisia*: According to Amar [8:163], the identification of the Biblical La’ana ((לענה is of “a low probability”. He also noted several other candidates: *Ecballium elaterium* (L.) A. Rich*., Citrullus colocynthis* L., and *Balanites aegyptiaca* (L.) Del.. All of these species are known as important medicinal plants in the ancient Fertile Crescent (*Artemisia* spp. [129,130]); *E. elaterium* [rev. 58]; *C. colocynthis* [131] and *B. aegyptiaca* [132]. Feliks [6:200] opines: “It is common to identify *La*’*ana* as plants from the genus *Artemisia* which contain bitter juice.” Zohary [7:184] identified two citations (Jeremiah 23:15 and Amos 5:7 as *Artemisia herba-alba* Asso*,* but “*La’ana*” appears in at least six more citations (Deuteronomy 29:18, Job 30:4, Proverbs 5:4, Lamentations 3:15 and 19, Hosea 10:4, Amos 6:12). The wormwood mentioned in Revelations 8:11 appears as “*Apsinthos*” in the Greek version. Padosch et al. [133] commented: “The Greek equivalent to “*Apsinthos*” is used as a name for a star that fell into the waters and turned them bitter. The Greek word “*Apsinthion*”—undrinkable—is most probably the ancestor of the word “absinthe.” The Talmud (Abodah Zara 30a) mentions preparing a special “Apsintin wine,” which is still produced today [133]. Thus, we prefer to treat “*La’ana*” as *Artemisia* spp., especially *A. herba alba*, which is known as a common medicinal plant in the Middle East and North Africa [134–136]. *A. absinthium* L. is also well known as a medicinal plant [139] and the wide use of *A. absinthium* in Egypt and Mesopotamia (Table 1). *B. aegyptiaca* seems confined to rare oases [137] and *E. elaterium* is rarely mentioned in ancient sources from the Holy Land [58].

## Conclusions

All our suggested BMPs are known as such also in Ancient Egypt and/or Mesopotamia (Tables 1–4). Explicit evidence for use of medicinal plants is very rare in the Bible as well as in the Jewish post-Biblical writings. The comparison to adjacent ancient civilizations (in time and space) enables us to reconstruct the suggested list of BMP’s. Examination of our list shows that all the plants in our suggested list are in continuous medicinal use in the Middle East down generations [138–139] and are used in the Holy Land today [138–141].

Shakya [142] published a review, “Medicinal plants: future source of new drugs.” His “top 25 bioactive compounds of medicinal plants” include *Ricinus communis, Piper nigrum,* Aloe vera, *Nigella sativa, Artemisia absinthium*, and *Allium sativum*. This list accounts for 24% of our suggested list of Biblical Medicinal Plants. As once spoken by King Solomon, “That which has been is what will be, That which is done is what will be done, And there is nothing new under the sun” (Ecclesiastes 1:9**).**

## Data Availability

Data sharing is not applicable to this article as no datasets were generated or analyzed during the current study.

## Abbreviations

AR: Archaeological evidence from the Holy Land
B: Old Testament
BT: Egypt
M: Mishna
NT: New Testament

## References

[1] Duke JA (1983). Medicinal Plants of the Bible.

[2] Jacob W, Jacob I, Jacob W (1993). Medicinal plants of the Bible: Another view. The healing past: pharmaceuticals in the Biblical and Rabbinic world.

[3] Löw I. Die Flora der Juden. Alexander Kohut Memorial Foundation:Wien - Leipzig. IV. Vols. 1924-1938.

[4] Moldenke HN, Moldenke AL (1952). Plants of the Bible.

[5] Feldmann U (1956). Plants of the Bible.

[6] Felix J (1957). Plant world of the Bible.

[7] Zohary M (1982). Plants of the Bible.

[8] Amar Z (2012). Plants of the Bible.

[9] Włodarczyk Z (2007). Review of plant species cited in the Bible. Folia Horticulturae..

[10] Tristram HB (1877). The natural history of the Bible: Being a review of the physical geography, geology, and meteorology of the holy land, with a description of every animal and plant mentioned in Holy Scripture.

[11] Henslow G (1896). The plants of the Bible.

[12] Hepper FN (1992). Illustrated encyclopedia of Bible plants.

[13] Hepper FN (1992). Baker encyclopedia of Bible plants: flowers and trees, fruits and vegetables.

[14] Maillat J, Maillat S (1999). Les plantes dans la Bible.

[15] Musselman LJ (2007). Figs, dates, laurel, and myrrh: plants of the Bible and the Quran.

[16] Musselman LJ (2012). A dictionary of Bible plants.

[17] Jensen HA. Plant world of the Bible. Bloomington: Author House; 2012.

[18] Rosner F (2000). Encyclopedia of medicine in the Bible and the Talmud.

[19] Borchardt JK (2002). The beginnings of drug therapy: Ancient Mesopotamian medicine. Drug News Prospect..

[20] Campbell-Thompson R (1949). A Dictionary of Assyrian Botany.

[21] Von Deines H, Grapow H (1959). Wörterbuch der ägyptischen Drogennamen.

[22] Pommerening T. Heilkundlische Texte aus dem Alten Ägypten: Vorschlag zur Kommentierung und Übersetzung. In: Imhausen A, Pommerening T, editors. Translating writings of early scholars in the Ancient Near East, Egypt, Greece and Rome: methodological aspects with examples. Berlin – New York: Walter de Gruyter; 2016. p. 175-280.

[23] Böck B, Kleber K, Neumann G, Paulus S (2018). Zur Weitergabe und Verbreitung altmesopotamischen medizinischen Wissens. Grenzüberschreitungen: Studien zur Kulturgeschichte des Alten Orients.

[24] Oppenheim AL (1964). Chicago Assyrian dictionary. Chicago: Oriental Institute Chicago.

[25] Soden von W. Akkadisches Handwörterbuch. 2 I-III. Wiesbaden: Otto Harrassowitz; 1972-1985.

[26] Westendorf W (1999). Handbuch der altägyptischen Medizin.

[27] Geller MJ (2005). Renal and Rectal Disease Texts.

[28] Crowfoot GMH, Baldensperger L (1932). From cedar to hyssop.

[29] Niebuhr A (1901). The ancient east No. II. The Tell El Amarna period. The relations of Egypt and western Asia in the fifteenth century B.C. according to the Tel El Amarna tablets by Carl Niebuhr. Translated Hutchinson J.

[30] Langgut D, Shahack-Gross R, Arie E, Namdar D, Amrani A, Le Bailly M, Finkelstein I (2016). Micro-archaeological indicators for identifying ancient cess deposits: An example from Late Bronze Age Megiddo. Israel. Journal of Archaeological Science: Reports..

[31] Weinstein-Evron M., Bratenkov S., Rosenberg D. A preliminary palynological analysis of Iron Age grinding stones from Tel Megiddo. In: Finkelstein I., Mario A., Martin S., Matthew J., Adams J., editors. Megiddo VI, The 2010–2014 Seasons. Chapter 50. In press.

[32] Dafni A, Khatib SA (2017). Plants, demons and wonders: Plants folklore in the Holy Land.

[33] Koh A., Yasur-Landau A., Cline EH. Characterizing a Middle Bronze palatial wine cellar from Tel Kabri, Israel. PloS One, 2014; 9(8), p.e106406. https://journa.ls.plos.org/plosone/article?id=10.1371/journal.pone.0106406 **(**visited 13.4.16) 10.1371/journal.pone.0106406 PMC4146609 25162228

[34] McGovern PE., Luley BP., Rovira N., Mirzoian A., Callahan MP., Smith KE., Hall GR., Davidson T., Henkin JM. Beginning of viniculture in France. Proceedings of the National Academy of Sciences. 2013; 110:10147-10152. https://www.pnas.org/content/pnas/110/25/10147.full.pdf **(**visited 13.4.16) 10.1073/pnas.1216126110 PMC3690885 23733937

[35] Namdar D, Gilboa A, Neumann R, Finkelstein I, Weiner S (2013). Cinnamaldehyde in early iron age Phoenician flasks raises the possibility of Levantine trade with South East Asia. Mediterranean Archaeology & Archaeometry..

[36] Kislev ME, Simchoni O, Melamed Y, Maroz L (2011). Flax seed production: evidence from the early Iron Age site of Tel Beth-Shean, Israel and from written sources. Vegetation History and Archaeobotany..

[37] Weiss E, Kislev ME (2004). Plant remains as indicators for economic activity: a case study from Iron Age Ashkelon. J Archaeol Sci.

[38] Harrison RK (1966). Healing herbs of the Bible.

[39] Byl SA. The essence and use of perfume in ancient Egypt. MA Thesis. 2012. Ancient Near Eastern Studies. University of South Africa.

[40] Ghalioungui P (1973). The house of life: magic and medicine in ancient Egypt.

[41] Nunn JF (2002). Ancient Egyptian Medicine.

[42] Böck B. Babylonische Divination und Magie als Ausdruck der Denkstrukturen des altmesopotamischen Menschen. In: Renger J., editor. Babylon: Focus mesopotamischer Geschichte, Wiege früher Gelehrsamkeit, Mythos in der Moderne. 2. Internationales Colloquium der Deutschen Saarbrücken: Orient-Gesellschaft; 1999. p 409-425.

[43] Ritner RK (1993). The mechanics of ancient Egyptian magical practice. Studies in Ancient Oriental Civilization 54.

[44] Böck B. The healing goddess Gula. Towards an understanding of ancient Babylonian medicine. Leiden–Boston: Brill; 2014.

[45] Geller MJ (2010). Ancient Babylonian medicine: theory and practice.

[46] Abusch T. Exorcism. I. Ancient Near East and Hebrew Bible/Old Testament. In: Allison DC. Jr., et al., editors. The Encyclopedia of the Bible and its reception. Berlin and Boston: Verlag Walter de Gruyter. 2014; 8: 513-519.

[47] Van der Toorn K. Sin and sanction in the Israel and Mesopotamia. Assen: an Gorcum. Studia Semitica Neerlandica. 22. 1985.

[48] Krymow V (2002). Healing plants of the Bible: history, lore and meditations.

[49] Kubo S (1971). A Reader's Greek-English lexicon of the New Testament.

[50] Vine WE (2015). Vines expository dictionary of New Testament words.

[51] Schonfield HJ (1985). The Original New Testament. Editor and translator.

[52] Feldmann U. Plants of the Mishna. Dvir: Tel Aviv; n.d. (In Hebrew)

[53] Kerner R. Medical materials and their use during the early Roman Era in Israel. PhD thesis. Martin Department of the Land of Israel Studies and Archaeology. Ramat Gan: Bar Ilan University, Israel. 2006.

[54] Budge EAW. The divine origin of the craft of the herbalist. London: Culpeper House;1928. https://ia800804.us.archive.org/18/items/b29826603/b29826603.pdf **(**visited 18.4.16)

[55] Falk JD (1966). The plants of Mari and Ugarit with special reference to the Hebrew Bible. PhD Dissertation.

[56] Scurlock J, May NN (2012). Getting smashed at the victory celebration, or what happened to Esarhaddon’s so-called vassal treaties and why. Iconoclasm and text destruction in the ancient Near East and beyond.

[57] Waniakowa J (2007). Mandragora and Belladonna–the names of two magic plants. Studia Linguistica, Universitatis Iagellonicae Cracoviensis..

[58] Dafni A (2013). Benítez GC., Blanché C., Rammón-Laca L., Petanidou T., Aytaç B., Horvat M., Lucchese F., Geva-Kleinberger. A. The etymological, ecological, historical and ethnobotanical roots of the vernacular names of *Ecballium elaterium* (L.) Rich. (Squirting cucumber). The Journal of Ethnobiology and Traditional Medicine. Photon..

[59] Riddle JM. Introduction. In: Jacob I., Jacob W. editors. The Healing Past: pharmaceuticals in the Biblical and Rabbinic world. Leiden: Brill; 1993. Pp. Xi-Xv.

[60] Attia A, Buisson G (2012). BAM 1 et consorts en transcription. Journal des Medecines Cuneiformes..

[61] Scurlock JA. Proposal for identification of a missing plant: Kamantu/ Ú ÁB. DUḪ= *Lawsonia inermis* L. " henna". Wiener Zeitschrift für die Kunde des Morgenlandes. 2007: 97; 491-520.

[62] Heessel NP. Reading and interpreting medical cuneiform texts – methods and problems”. Le Journal des Médecines Cunéiformes. 2004:3; 2–9.

[63] Geller MJ (1995). Reviews of books-The healing past: pharmaceuticals in the Biblical and Rabbinic world edited by Irene Jacob and Walter Jacob. Journal of the American Oriental Society..

[64] Germer R, Jacob I, Jacob W (1993). Ancient Egyptian pharmaceutical plants and the eastern Mediterranean. The healing past: pharmaceuticals in the Biblical and Rabbinic world.

[65] Manniche L. An ancient Egyptian herbal. Austin: University of Texas Press and London: British Museum. 1989.

[66] Du Toit JS, Naudé JA (2005). Lost in translation: designation, identification and classification of flora in translated Biblical Hebrew texts. Journal of Northwest Semitic Languages..

[67] Steinsaltz A (1967). Talmud Bavli (Hebrew translation).

[68] Aboelsoud NH (2010). Herbal medicine in ancient Egypt. Journal of Medicinal Plants Research..

[69] Leake CD (1952). The old Egyptian medical papyri.

[70] Germer R. Untersuchungen über Arzneipflanzen im alten Ägypten (PhD. Thesis). Hamburg; 1979.

[71] Tavernier J (2008). KADP 36: Inventory, plant list, or lexical exercise? In: Biggs RD., Meyer J., Roth MT., editors. Proceedings of the *51st* Rencontre Assyriologique Internationale. Chicago: The Oriental Institute of the University of Chicago. Studies in Ancient Oriental Civilizations..

[72] Fincke JC. Cuneiform tablets on eye diseases: Babylonian sources in relation to the series diš na igiII-šú gig. In Attia A., Buisson G., editors. Advances in Mesopotamian medicine from Hammurabi to Hippocrates: Proceedings of the International Conference. Oeil Malade et Mauvais Oeil. Paris Collège de France. Brill; 2009. p 79-104.

[73] Giusfredi F. The Akkadian medical text KUB 37.1. Altorientalische Forschungen. 2012; 39:49-63.

[74] Amar Z (2012). The Afarsemon from Ein Gedi and the story of Massada. Judea and Samaria Studies..

[75] Bryan PW (1930). The Papyrus Ebers.

[76] Purnima BM, Kothiyal P (2015). A review article on phytochemistry and pharmacological profiles of *Nardostachys jatamansi* DC-medicinal herb. Journal of Pharmacognosy and Phytochemistry..

[77] Balakumbahan R, Rajamani K, Kumanan K (2010). *Acorus calamus*: an overview. Journal of Medicinal Plants Research..

[78] Scurlock J (2014). Sourcebook for ancient Mesopotamian medicine (writings from the ancient world).

[79] Powell MA. Obst und Gemüse. A. I. Mesopotamien. In: Dietz O. Edzard (Hg.). Reallexikon der Assyriologie und Vorderasiatischen Archäologie. Berlin-New York: de Gruyter; 2003–2005; 10:13–22.

[80] Heessel NP, Al-Rawi FN (2003). Tablets from the Sippar library XII. A medical therapeutic text. Iraq.

[81] Böck B. On medical technology in Ancient Mesopotamia. In: Attia A, Buisson G, editors. Advances in Mesopotamian medicine: From Hammurabi to Hippocrates. Leiden – Boston: Brill; 2009. Pp. 105-128.

[82] Parpola S (1983). Letters from Assyrian scholars to the kings Esarhaddon and Assurbanipal.

[83] Salin S (2016). Transmission and interpretation of therapeutic texts. Šumma amēlu muḫḫašu umma ukāl: a case study. Distant Worlds Journal.

[84] Langgut D, Gadot Y, Porat N (2013). Lipschits. O. Fossil pollen reveals the secrets of the royal Persian garden at Ramat Rahel, Jerusalem. Palynology..

[85] Langgut D (2015). Prestigious fruit trees in ancient Israel: first palynological evidence for growing *Juglans regia* and *Citrus medica*. Israel Journal of Plant Sciences.

[86] Jursa M. Die Kralle des Meeres and andere Aromata. Philologisches und Historisches zwischen Anatolien und Sokotra: analecta semitica in memoriam Alexander Sima, 2009. Pp. 147-180.

[87] Heessel NP. The Babylonian physician Rabâ-ša-Marduk. Another look at physicians and exorcists in the ancient Near East. In: Attia A., Buisson G., editors. Advances in Mesopotamian Medicine: From Hammurabi to Hippocrates. Leiden – Boston: Brill; 2009. Pp. 13-28.

[88] Kinnier-Wilson JK (2005). The Assyrian pharmaceutical series URU. AN. NA: MAŠTAKAL. Journal of Near Eastern Studies..

[89] Levey M (1955). Ancient chemical technology in a Sumerian pharmacological tablet. Journal Chemical Education..

[90] Limet H (1982). Les Sumériens et les plantes. Horn: Verlag Ferdinand und Söhne GmbH. Beiheft Archiv für Orientforschung.

[91] Bottéro*, J. Mesopotamia:* Writing, reasoning, and the gods. Chicago: University of Chicago Press; 1995.

[92] Foster JT., Allan GJ., Chan AP., Rabinowicz PD., Ravel J., Jackson PJ., Keim P. Single nucleotide polymorphisms for assessing genetic diversity in castor bean (*Ricinus communis*). BMC Plant Biology. 2010.10(1). https://bmcplantbiol.biomedcentral.com/articles/10.1186/1471-2229-10-13 **(**visited 16.4.16) 10.1186/1471-2229-10-13 PMC2832895 20082707

[93] Waetzoldt H (1992). "Rohr" und dessen Verwendungsweisen anhand der neusumerischen Texte aus Umma. Cambridge: Sumerian Agriculture Group, Faculty of Oriental Studies. BSA.

[94] Stol M, Edzard DO (1980). Leder (industrie). Reallexikon der Assyriologie und Vorderasiatischen Archäologie.

[95] Grace O, Buerki S, Symonds MR, Forest F, van Wyk AE, Smith GF, Klopper RR, Bjorå CS, Neale S, Demissew S, Simmonds MS. Evolutionary history and leaf succulence as explanations for medicinal use in aloes and the global popularity of *Aloe vera*. BMC Evolutionary Biology. 2015; 15: (1) 29. https://bmcevolbiol.biomedcentral.com/articles/10.1186/s12862-015-0291-7 **(**visited 13.7.16) 10.1186/s12862-015-0291-7 PMC4342203 25879886

[96] Zeist Van W. Some notes on second millennium BC plant cultivation in Syrian Jazira. In: Gasch H., Tarnet M., editors. Cinquante-deux Réflexions sur le Proche-Orient Ancien, Offertes en Hommage à Léon De Meyer. Leuven: Peeters; 1994. Pp. 541-553.

[97] Chapman MA., Burke JM. DNA sequence diversity and the origin of cultivated safflower (*Carthamus tinctorius* L.; Asteraceae). BMC Plant Biology. 2007; 7:(1), p.60. https://bmcplantbiol.biomedcentral.com/articles/10.1186/1471-2229-7-60 **(**visited 3.4.16) 10.1186/1471-2229-7-60 PMC2241619 17986334

[98] Ross IA (2005). Medicinal plants of the world Vol 3.

[99] Parsa A (1960). Medicinal plants and drugs of plant origin in Iran. IV. Plant Foods for Human Nutrition (formerly Qualitas Plantarum).

[100] Haussperger M (1970). Die Krankheiten des Verdauungstraktes. Die Welt des Orients. 2002; 1:33-73. Hui-Lin L. The origin of cultivated plants in Southeast Asia. Economic Botany..

[101] Farber W. Altassyrisch addahšū und hazuannū, oder von Safran, Fenchel, Zwiebeln und Salat. Zeitschrift für Assyriologie und Vorderasiatische Archäologie. 1991;81:234–42.

[102] Weinstein B (2000). Biblical evidence of spice trade between India and the land of Israel: a historical analysis. Indian Historical Review..

[103] Ben-Yehoshua S, Borowitz C, Hanuš LO (2012). Frankincense, Myrrh, and Balm of Gilead: ancient spices of southern Arabia and Judea. Horticultural Reviews..

[104] Gilboa A, Namdar D (2015). On the beginnings of south Asian spice trade with the Mediterranean region: a review. Radiocarbon..

[105] Bisset NG, Bruhn JG, Curto S, Holmstedt B, Nyman U, Zenk MH. Was opium known in 18th dynasty ancient Egypt? An examination of materials from the tomb of the chief royal architect Kha. Journal of Ethnopharmacology. 1994; 41: 99-106.Merrillees RS. Highs and lows in the Holy Land: opium in Biblical times. Eretz-Israel: Archaeological, Historical and Geographical. Studies. 1989:148–53. 10.1016/0378-8741(94)90064-7 8170167

[107] Merlin MD (2003). Archaeological evidence for the tradition of psychoactive plant use in the old world. Economic Botany..

[108] Chovanec Z, Bunimovitz S, Lederman Z (2015). Is there opium here? Analysis of Cypriote base ring juglets from Tel Beth-Shemesh. Israel. Mediterranean Archaeology and Archaeometry..

[109] Feinbrun-Dothan N, Danin A (1991). Analytical flora of the land of Israel.

[110] Dafni A, Yaniv Z, Palevitch D (1984). Ethnobotanical survey of medicinal plants in northern Israel. Journal of Ethnopharmacology..

[111] Lev E, Amar Z (2002). Ethnic medicinal substances of the land of Israel.

[112] Postgate N (1992). Trees and timbers in the Assyrian texts. Bulletin on Sumerian Agriculture..

[113] Liphschitz N (1987). *Ceratonia siliqua* in Israel: An ancient element or a newcomer?. Israel Journal of Botany..

[114] Kislev M. The History of the Carob in Israel. Halamish. 1988; 6:20-30. (Hebrew).

[115] Zohary D (2002). Domestication of the carob (*Ceratonia siliqua* L.). Israel J Plant Sci.

[116] Albert RM., Weiner S. Study of phytoliths in prehistoric ash layers from Kebara and Tabun caves using a quantitative approach. Phytoliths. In: Meunier JD., Colin F., editors. Applications in earth sciences and human history. Abington: AA Balkema; 2001 Pp. 251-266.

[117] Galili E., Kolska-Horwitz L., Rosen B., Eshed V. Submerged pottery neolithic settlements off the Mediterranean coast of Israel. In: Bailey G., Harff J., Sakellariou D., editors. Under the sea: archaeology and palaeolandscapes of the continental shelf. Springer: Coastal Research Library 20. 2017: 105–130.

[118] Melamed Y (2002). Chalcolithic and Hellenistic plant remains from cave V/49. Atiqot..

[119] Aharonovich S, Sharon G, Weinstein-Evron M (2014). Palynological investigations at the middle Palaeolithic site of Nahal Mahanayeem outlet. Israel. Quaternary International..

[120] American Holy Land Expedition. Bible witnesses from Bible Land. Joppa and Jerusalem. 1874. https://books.google.co.il/books?id=VgkYnMU2uA0C&dq=editions:DQ4XKIstCR0C&source=gbs_navlinks_s **(**visited 13.2.15)

[121] Felix Y (1994). Fruit trees in the Bible and Talmudic literature.

[122] Hadi MY, Hameed IH, Ibraheam IA (2017). *Ceratonia siliqua*: characterization, pharmaceutical products and analysis of bioactive compounds: a review. Research Journal of Pharmacy and Technology..

[123] Hansman J (1976). Gilgamesh, Humbaba and the land of the Erin-trees. Iraq..

[124] Sherwin S (2003). In search of trees: Isaiah XLIV 14 and its implications. Vetus Testamentum..

[125] Lawrence PJ (2004). Bĕrôš—A study in translational inconsistency. The Bible Translator..

[126] Löw I (1881). Aramaeische pflanzennamen.

[127] Prajapati RP., Kalariya M., Parmar SK., Sheth NR. Phytochemical and pharmacological review of *Lagenaria sicereria*. J Ayurveda Integ Med. 2010; 1: 266-272. https://www.ncbi.nlm.nih.gov/pmc/articles/PMC3117318/. **(**visited 1.8.16) 10.4103/0975-9476.74431 PMC3117318 21731373

[128] Jacob I, Jacob I, Jacob W (1993). *Ricinus Communis*—The miracle tree through four thousand years. The healing past: pharmaceuticals in the Biblical and Rabbinic world.

[129] Abad MJ., Bedoya LM., Apaza L., Bermejo P. The *Artemisia* L. genus: a review of bioactive essential oils. Molecules. 2012; 17: 2542-2566. https://www.mdpi.com/1420-3049/17/3/2542 **(**visited 23.5.16) 10.3390/molecules17032542 PMC6268508 22388966

[130] Al-Snafi AE (2015). The pharmacological importance of *Artemisia campestris*-a review. Asian Journal of Pharmaceutical Research..

[131] Rahimi R, Amin G, Ardekani MRS (2012). A review on *Citrullus colocynthis* Schrad.: from traditional Iranian medicine to modern phytotherapy. The Journal of Alternative and Complementary Medicine..

[132] Chothani DL, Vaghasiya HU (2011). A review on *Balanites aegyptiaca* Del (desert date): phytochemical constituents, traditional uses, and pharmacological activity. Pharmacognosy Reviews.

[133] Padosch SA., Lachenmeier DW., Kröner LU. Absinthism: a fictitious 19th century syndrome with present impact. Substance Abuse Treatment, Prevention, and Policy. 2006;1:14 https://substanceabusepolicy.biomedcentral.com/articles/10.1186/1747-597X-1-14 **(**visited 13.4.16) 10.1186/1747-597X-1-14 PMC1475830 16722551

[134] Khlifi D, Sghaier RM, Amouri S, Laouini D, Hamdi M, Bouajila J (2013). Composition and anti-oxidant, anti-cancer and anti-inflammatory activities of *Artemisia herba*-*alba*, *Ruta chalpensis* L. and *Peganum harmala* L. Food and Chemical Toxicology..

[135] Akrout A, El Jani H, Amouri S, Neffati M (2009). Screening of antiradical and antibacterial activities of essential oils of *Artemisia campestris* L., *Artemisia herba alba* Asso, & *Thymus capitatus* Hoff. et Link. growing wild in the southern of Tunisia. Recent Research in Science and Technology..

[136] Goud BJ, Swamy BC (2015). A review on history, controversy, traditional use, ethnobotany, phytochemistry and pharmacology of *Artemisia absinthium* Linn. International Journal of Advanced Research in Engineering and Applied Science..

[137] Zohary M (1962). Plant life of Palestine: Israel and Jordan.

[138] Lev E (2002). Reconstructed materia medica of the Medieval and Ottoman al-Sham. Journal of Ethnopharmacology..

[139] Lev E (2007). Practical materia medica of the medieval Eastern Mediterranean according to the Cairo Genizah.

[140] Lev E (2015). Botanical view of the use of plants in medieval medicine in the eastern Mediterranean according to the Cairo Genizah. Israel Journal of Plant Sciences..

[141] Amar Z, Lev E (2016). Arabian drugs in Medieval Mediterranean medicine.

[142] Shakya AK (2016). Medicinal plants: future source of new drugs. International Journal of Herbal Medicine..

[143] Zohary D, Hopf M, Weiss E (2012). Domestication of Plants in the Old World: the origin and spread of domesticated plants in Southwest Asia, Europe, and the Mediterranean Basin.

